# Burden of COVID-19 in immunocompromised patients in Germany: a retrospective, observational Study on Health Insurance Data from 2021 to 2022

**DOI:** 10.1007/s15010-025-02516-w

**Published:** 2025-04-11

**Authors:** Julia Weinmann-Menke, Clemens-Martin Wendtner, Dennis Häckl, Vanessa Lohe, Phi Long Dang, Fungwe Jah, Nikolaus Kneidinger

**Affiliations:** 1 Department of Medicine and Research Center for Immunotherapy (FZI), University Medical Center of the Johannes Gutenberg University, Mainz, Germany; 2 Med. Klinik III, Ludwig-Maximilians-Universität (LMU), Munich, Germany; 3 Universität Leipzig, Lehrstuhl Für Health Economics and Management, Leipzig, Germany; 4 Wissenschaftliches Institut Für Gesundheitsökonomie Und Gesundheitssystemforschung (WIG2) GmbH, Leipzig, Germany; 5 AstraZeneca GmbH, Hamburg, Germany; 6 Department of Medicine V, LMU University Hospital, LMU Munich, Comprehensive Pneumology Center Munich (CPC-M), German Center for Lung Research (DZL), Munich, Germany; 7 Division of Pulmonology, Department of Internal Medicine, Lung Research Cluster, Medical University of Graz, Graz, Austria

**Keywords:** COVID-19, Immunocompromised, Claims data, Hematological malignancies, Chronic kidney disease, Oncology

## Abstract

**Purpose:**

Patients with COVID-19 and immunocompromising conditions are threatened with higher morbidity, mortality and a greater economic burden than immunocompetent persons due to an inadequate immune response to infection and vaccination. Health and economic COVID-19 outcomes in 2021 and 2022, a period during which vaccines became available gradually, were investigated.

**Methods:**

This retrospective observational study was based on statutory health insurance (SHI) claims data of approximately 2.7 million insurees each of 2021 and 2022, extrapolated to the overall German SHI population. An immunocompromised group was defined via several risk factors. COVID-19-related outcomes were compared to a group without risk factors (immunocompetent group).

**Results:**

In both years, COVID-19-associated hospitalizations were significantly elevated in the immunocompromised group (33.11% vs 7.88% in 2021, 19.25% vs 2.21% in 2022), as were ICU admission (9.17% vs 1.75% and 3.94% vs 0.32%), mortality (9.70% vs 1.62% and 3.42% vs 0.30%), and average costs for hospitalizations (17,966 € vs 12,769 € and 16,640 € vs 10,853 €). Hospitalization/intensive care unit (ICU) admission rates and COVID-19 associated mortality decreased from 2021 to 2022 in both groups, but more prominently in the immunocompetent group. Consequently, the gap between both groups increased.

**Conclusion:**

From 2021 to 2022, the health and economic burden of COVID-19 remained substantially elevated in the immunocompromised group, despite availability of vaccines and authorized treatments.

**Supplementary Information:**

The online version contains supplementary material available at 10.1007/s15010-025-02516-w.

## Introduction

From January 2020 to April 2024, the spread of SARS-CoV-2 has caused over 38.8 million infections and more than 180,000 deaths in Germany [1], resulting in immense strain on the public health system. Patients with an impaired immune response to either infection or vaccination are threatened with higher morbidity and mortality due to COVID-19, as well as significantly higher hospitalization costs [2–6].

Häckl et al. [6] previously evaluated COVID-19-associated hospitalizations, mortality risk and economic burden in several groups identified as being at risk of not developing a sufficient immune response to infection due to underlying conditions or treatments, based on an analysis of SHI claims data from 2020 [6]. The study observed a high vulnerability of the immunocompromised group, particularly patients with hematological malignancies, regarding health and economic outcomes. As vaccines were not available in 2020, the potential benefit of vaccination for the investigated groups could not be assessed in comparison to immunocompetent individuals.

Initial vaccination campaigns targeted vulnerable populations, including the elderly and immunocompromised individuals. Therefore, vaccination rates and timing differed between the respective populations: While the vaccination rate of adults aged 18–59 years reached 50% in calendar week 29 in 2021, that of the age group > 60 years (which was prioritized similarly as immunocompromised individuals) reached 50% already in calendar week 23. At the end of 2021, the age group > 60 years had a vaccination rate > 85% compared to 70% in the general population; by end of 2022, primary vaccination rates were 90% and 80%, respectively. In 2022, first booster rates were 85% and 65% and second booster rates were 35% and 5%, respectively [7].

The present study analyzes two corresponding datasets covering the years 2021 and 2022 with the aim of investigating the development of COVID-19 associated morbidity and mortality in immunocompromised individuals compared to an immunocompetent group over a period during which vaccines became available gradually.

## Methods

This retrospective observational study is based on SHI data accessed through the research database of the independent research institute “Wissenschaftliches Institut für Gesundheitsökonomie und Gesundheitssystemforschung” (WIG2). The evaluated time periods spanned January 1 to December 31 2021 and 2022, respectively. Anonymized demographic data and longitudinal claims data for in-hospital and outpatient treatments, as well as costs for reimbursed medicinal products of over 4 million SHI insurees were extracted for both regarded periods; after selection according to the inclusion criteria (see below), the study sample contained approximately 2.7 million subjects for each year. The samples were representative of the whole German SHI population in terms of age, gender and morbidity; numbers of insurees within the defined groups were then extrapolated to the entire German SHI population using KM6-statistics [24], standardized by age and gender.

For inclusion into the study, subjects had to be at least 12 years old and to be insured at the same insurance scheme over the entire observational period (or died after having been insured for at least one day). Retrieved data for each year were stratified into a group considered to be at risk of not developing an adequate immune response to either vaccination or infection according to several risk factors (the immunocompromised group) and an immunocompetent group without these risk factors. Details on how the risk factors were operationalized for analysis are provided in the supplementary Table S1, listing the codes used for data extraction from SHI data. Risk factors were autoimmune disorder under immunosuppressive treatment [8–10], chronic kidney disease (CKD) requiring dialysis [11, 12], hepatic fibrosis and cirrhosis [13, 14], cancer under therapy [15–17], hematological malignancies under therapy [18–20], organ transplant [21, 22], or primary/secondary immunodeficiencies [3, 23]. Subgroup analyses for the described immunocompromised groups were also performed. In addition to the codes required for the definition of the immunocompromised groups, Table S1 also lists the codes defining outpatient COVID-19 diagnosis and/or hospitalization for COVID-19 to determine COVID-19 related interactions with the healthcare system and the associated economic impact.

Differences between the immunocompromised and immunocompetent groups (including subgroups) with regards to COVID-19 incidence, hospitalization for COVID-19, hospitalization with ICU admission and mortality were investigated by calculation of the relative risk (RR), corresponding 95% confidence intervals (95% CI), t-test for independent samples and Chi-square tests. Two-sided p-values ≤ 0.05 were defined as statistically significant. The analyses were performed using Microsoft SQL Server Management Studio (17.4) and R Studio Version 4.2.0. Data were plotted using R Studio Version 4.2.0 with packages forestplot, dplyr, tidyr, readxl [25].

## Results

### Population sizes and characteristics

For all investigated groups and subgroups, the counts in the investigated sample as well as the number extrapolated to the entire SHI population are depicted in Table 1. The extrapolated number of subjects defined as immunocompromised was composed of 1,846,763 SHI-insurees (2.81% of the SHI population) in 2021, and 1,726,071 (2.68%) in 2022. The immunocompetent group comprised 63,956,451 (97.19%) and 64,360,068 (97.39%) insurees, respectively. The proportion of females was similar between groups for both cohorts, while the average age was consistently higher in the immunocompromised group.

**Table 1:** Composition of the group at risk and the group not at risk

Evaluated groups							
	Period	Subjects (investigated sample)	Subjects (extrapolated to full SHI population)	% Female	Mean age (SD)	Proportion of subjects in SHI^a^	Proportion of subjects in IC group^a^
**Immunocompetent (control group)**	2021	2,650,319	63,956,451	52.1	48.9 (20.0)	97.2%	N/A
	2022	2,633,249	64,360,068	52.2	49.2 (20.0)	97.4%	N/A
**Immunocompromised**	2021	77,877	1,846,763	53.4	60.3 (16.4)	2.8%	100%
	2022	72,602	1,726,071	52.4	61.1 (16.3)	2.7%	100%
Autoimmune disorder under immunosuppressive treatment	2021	18,624	444,731	62.2	55.1 (15.0)	0.7%	24.1%
	2022	13,621	325,107	58.7	55.0 (14.9)	0.5%	18.8%
CKD requiring dialysis	2021	5,231	122,320	39.0	66.8 (15.0)	0.2%	6.6%
	2022	5,234	122,454	39.4	67.0 (14.9)	0.2%	7.1%
Hepatic fibrosis and cirrhosis	2021	14,395	329,876	42.6	63.6 (12.6)	0.5%	17.9%
	2022	14,396	330,718	42.6	64.0 (12.6)	0.5%	19.2%
Cancer under therapy	2021	31,760	756,441	53.8	66.2 (12.8)	1.2%	41.0%
	2022	31,847	758,461	53.8	[66.4;12.8]	1.2%	43.9%
Hematological malignancies under therapy^a^	2021	5,379	127,269	46.7	66.5 (14.7)	0.2%	6.9%
	2022	5,519	130,558	46.2	66.7 (14.8)	0.2%	7.6%
Organ transplant	2021	3,830	87,224	41.6	55.7 (15.3)	0.1%	4.7%
	2022	3,768	86,543	41.1	55.9 (15.5)	0.1%	5.0%
Primary and/or secondary immunodeficiencies	2021	10,855	265,397	59.1	47.4 (20.8)	0.4%	14.4%
	2022	9,946	246,994	59.7	47.7 (21.0)	0.4%	14.3%

The numbers of insurees within the defined groups were extrapolated to the entire SHI population using KM6 statistics, standardized by age and gender [26]

Abbreviations: *CKD* Chronic kidney disease, *SHI* statutory health insurance

^a^As patients with hematological malignancies are frequently treatmed with B-cell depleting therapies resulting in substantially lower seroconversion rates compared to patients with solid tumors [18, 19], a separate analysis of this subgroup is provided

### COVID-19 incidence and clinical outcomes

**Fig. 1:**
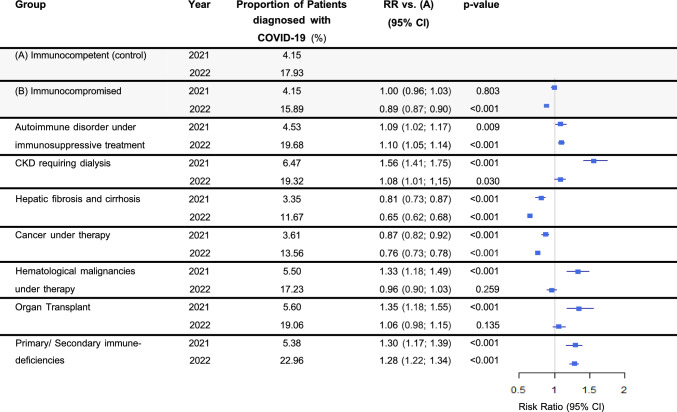
COVID-19 incidence. “Proportion” refers to the fraction of the respective risk group diagnosed with COVID-19 according to ICD-10 codes indicated in Supplementary Table S1. Abbreviations: *CI* Confidence interval, *CKD* Chronic kidney disease, *RR* Relative risk

COVID-19 incidence in both groups increased markedly between 2021 and 2022. While 2021 incidences had been identical in both groups (both 4.15%, Fig. 1), a significantly lower incidence was observed in the immunocompromised group compared to the immunocompetent group in the 2022 cohort (15.89% vs 17.93%, RR 0.89, p < 0.001).

**Fig. 2:**
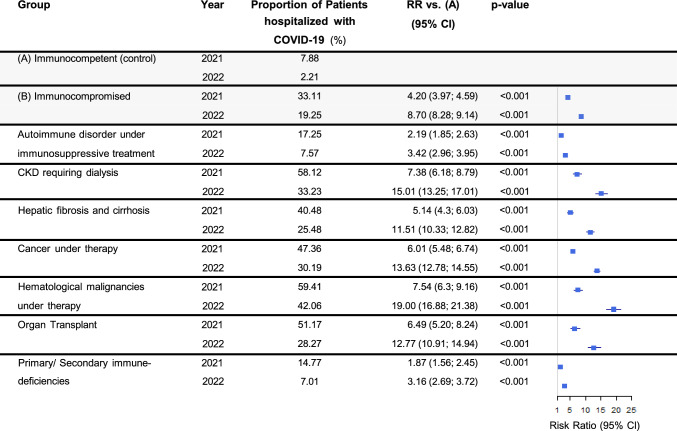
COVID-19-associated hospitalizations. “Proportion” refers to the fraction of COVID-19 patients within the indicated population that was hospitalized. Abbreviations: *CI* Confidence interval, *CKD* Chronic kidney disease, *RR* Relative risk

**Fig. 3:**
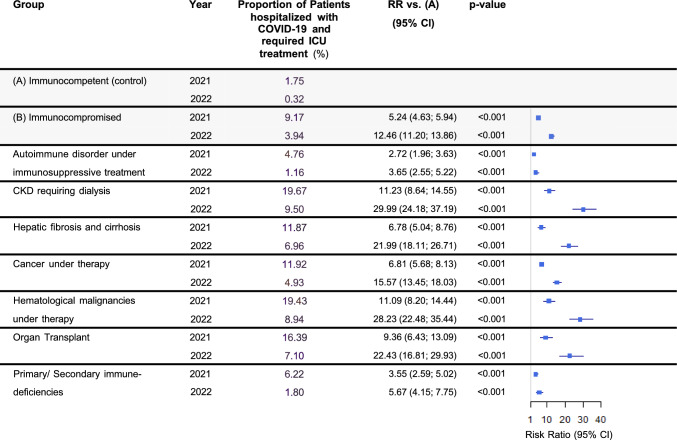
Intensive care treatment of COVID-19 patients. “Proportion” refers to the fraction of COVID-19 patients within the indicated population that was hospitalized and required intensive care treatment. Abbreviations: *CI* Confidence interval, *CKD* Chronic kidney disease, *RR* Relative risk

COVID-19-associated hospitalizations decreased for both groups between 2021 and 2022 (33.11% to 19.25% for the immunocompromised vs 7.88% to 2.21% for the immunocompetent group), while the RR for hospitalization was significantly higher for the immunocompromised group in both years (4.20 and 8.70, both p < 0.001, Fig. 2). Similarly, the risk for ICU admission decreased in both groups from 2021 to 2022 (9.17% to 3.94% for the immunocompromised group vs 1.75% to 0.32%), with a substantially elevated risk for the immunocompromised group (RR 5.24 and 12.46, both p < 0.001, Fig. 3).

**Fig. 4:**
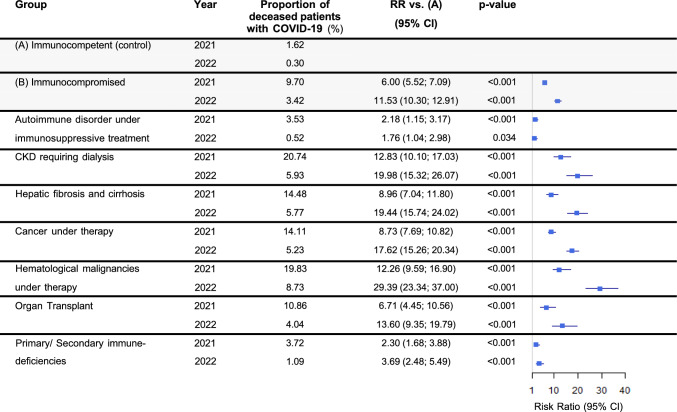
Mortality in COVID-19 patients. Abbreviations: *CI* Confidence interval, *CKD* Chronic kidney disease, *RR* Relative risk

Mortality rates for COVID-19 patients declined from 2021 to 2022 (9.70% to 3.42% for the immunocompromised vs 1.62% to 0.30% for the immunocompetent group) with a concomitant increase of RR for the immunocompromised group from 6.00 to 11.53 (both p < 0.001, Fig. 4).

Within the immunocompromised group, COVID-19-associated hospitalization, ICU admission and mortality were highest in patients with CKD requiring dialysis and hematological malignancies under therapy in both cohorts.

### Length of stay and costs of COVID-19 associated hospitalizations

**Fig. 5:**
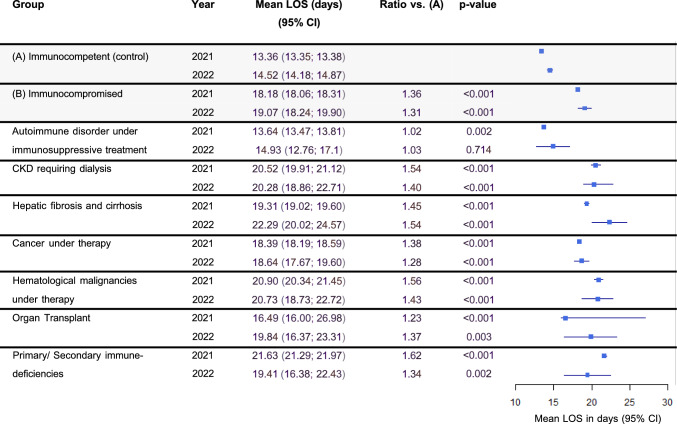
Length of hospital stays of COVID-19 patients. Abbreviations: *CI* Confidence interval, *CKD* Chronic kidney disease

The mean length of hospital stays remained relatively constant within groups between 2021 and 2022 (18.18 and 19.07 days in the immunocompromised group vs 13.36 and 14.52 days). In both periods, the immunocompromised group had longer stays on average (with a ratio of 1.36 and 1.31, respectively in the immunocompromised vs the immunocompetent group, Fig. 5). Likewise, costs of hospitalization were on average 41% and 53% higher for the immunocompromised group (17,966 € vs 12,769 € in 2021, and 16,640 € vs 10,853 € in 2022, Fig. 6).

**Fig. 6:**
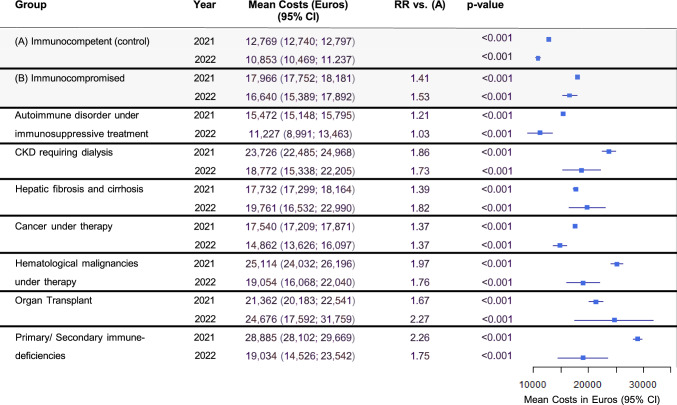
Costs of hospitalization of COVID-19 patients. Abbreviations: *CI* Confidence interval, *CKD* Chronic kidney disease

## Discussion

The presented study analyzed the health and economic burden of COVID-19 in several immunocompromised patient groups, who were considered as being at risk of not developing an adequate immune response to either COVID-19 infection or vaccination. This was done by evaluation of two sets of SHI claims data, one from 2021 and one from 2022. An analogous evaluation has previously been performed for the year 2020, before vaccines were available. The present study aimed at investigating the development of COVID-19 outcomes in the immunocompromised group over a time span during which vaccines became available. Due to lower seroconversion rates in the identified subgroups of immunocompromised patients [3, 8–23], a lower benefit than in the immunocompetent reference group was expected.

Apart from vaccine availability, several external factors differed between 2021/2022 and 2020: During the first COVID-19 wave in 2020, case numbers were still relatively low with 7-day incidences greater than 100/100,000 only reported in fall, while both 2021 and 2022 were full pandemic years with incidences peaking at almost 2,000/100,000 in March 2022 [7, 26, 27]. Further, new virus variants with higher transmissibility emerged: The wildtype strain dominating 2020 was replaced by the alpha variant in the beginning of 2021, while delta was the most prevalent variant during the fall wave and omicron during 2022 [28–30]. Morbidity and mortality associated with both alpha and delta are described as greater than wildtype [31, 32] with delta being more virulent [29, 31]. Disease severity and mortality rates reported for omicron in contrast are significantly lower, both in vaccinated and unvaccinated individuals [29, 32, 33]. A detailed quantitative comparison of global morbidity and mortality associated with the different variants is however challenging due to multiple confounding factors, such as varying rates of vaccination and immunity after previous infection across regions and during different stages of the pandemic [31–34]. In addition, compliance to isolation measures over time and patient groups presumably resulted in heterogeneous exposure rates.

The increase in overall COVID-19 incidence according to the SHI data sets from 2020 to 2022 is consistent with the global dynamics of the pandemic. Remarkably, the COVID-19 incidence in the immunocompromised group that had been significantly higher than that in the control group in 2020 (1.96% vs 1.62%, p < 0.001 [6]) was equal in 2021 (4.15% both) and decreased below that of the control group in 2022 (15.89% vs 17.93%, p < 0.001). Multifactorial causes may account for this development: While the higher incidence in 2020 may be assumed to relate to the impaired immune response of the immunocompromised group, early vaccination of immunocompromised individuals in 2021 likely compensated lower seroconversion rates, such that a larger part of the population was protected at the onset of the fall wave than in the control group, i.e. higher vaccination rates levelled out the lower response rates. In line with this assumption, patients with hepatic disease, a subgroup with relatively high seroconversion rates of 90% after second and 70% after third vaccination [13, 14] that was nevertheless prioritized in early vaccination campaigns, showed an incidence below group average in both years. Another factor impacting relative incidences, particularly in 2022 when incidences in the immunocompromised group fell below the control, may have been a more cautious lifestyle of subjects at risk, i.e. greater compliance with measures like social distancing, wearing of face masks, hand hygiene etc., after the relief of official restraints in spring 2022 [35], which was frequently observed in clinical practice.

COVID-19-associated hospitalization or ICU admission and mortality were significantly higher in the immunocompromised group in previous reports [6, 36] as well as in the presented data sets from 2021 and 2022; comparable data have been reported in retrospective cohort studies in the USA and the UK [28, 37]. The difference between groups is unsurprising, given not only the impaired immune response, but also a worse general condition on average due to the underlying condition and the potential for disease decompensation. In addition, preventive hospitalization due to the respective risk factors may have contributed to the increased COVID-19-associated hospitalization rate.

It is noteworthy that the risk of severe disease – reflected by hospitalization, ICU and mortality rates – decreased from 2021 to 2022 in both groups. A combination of vaccine availability and authorized treatments, immunity due to previous infection and, in 2022, the lower virulence of omicron compared to previously dominating variants may account for this development. Clearly, the effect was more pronounced in the immunocompetent group than in the immunocompromised group, such that the RR for all three parameters increased markedly.

Vaccination status per subject could not be assessed via the SHI data. Therefore, only indirect conclusions on the efficacy of vaccination in immunocompromised subjects compared to the control group can be drawn from the presented data. However, published data investigating the risk of severe COVID-19 stratified by vaccination status similarly report a significantly elevated risk of hospitalization, ICU admission and COVID-19 related death in immunocompromised subjects, both when vaccinated and unvaccinated groups are compared. In line with the presented data set, the authors concluded that protection by vaccination is reduced in the immunocompromised group [28, 38–42].

Hospital stays were consistently longer in the immunocompromised group (with a ratio of 1.36 for the immunocompromised vs immunocompetent group in 2021 and 1.31 in 2022).

Average treatment costs were substantially higher for the immunocompromised group in both years, corresponding to the increased ICU admission rate and on average longer hospital stays. Additional factors may have contributed, such as a worse general condition, decompensation of the underlying disease, comorbidities and additional treatment requirements.

Limitations of this study include the common restraints of claims data analyses, i.e. incorrect or incomplete coding. Moreover, coding for COVID-19 infection was restructured between 2020 and 2022. Post-COVID-19 state (ICD-10 code U09.9!) and multisystem inflammatory syndrome associated with COVID-19 (U10.9) were included in the 2022, but not the 2021 analysis (Supplementary Table S1). Besides, not all infections, particularly asymptomatic or very mild cases, were captured in this database, such that total infection rates were likely underestimated and the fraction of cases presenting with severe disease overestimated. Finally, the efficacy of vaccination in the prevention of severe COVID-19 in the immunocompromised group cannot conclusively be evaluated, due to multiple confounding factors and the inability to stratify the data set by vaccination status.

Overall, despite the availability of efficacious vaccines and treatments, the health and economic burden of COVID-19 in the immunocompromised group remained high from 2020/2021 to 2022 and substantially exceeded that of the immunocompetent group. While overall morbidity and mortality rates decreased substantially in both groups, likely due to the availability of vaccines and treatments as well as the reduced virulence of omicron, the effect was less pronounced in the immunocompromised group. Therefore, the gap in terms of health and economic outcomes between both groups increased over the regarded period. Additional protective measures need to be considered for this highly vulnerable population.

## Supplementary Information

Below is the link to the electronic supplementary material.

Supplementary file1 (DOCX 26 KB)

## Data Availability

The datasets generated during and/or analyzed during the current study are not publicly available due to data protection aspects. The data underlying this article will be shared at aggregate/population level upon reasonable request to the corresponding author.
