# Supplementary material for: Burden of COVID-19 in immunocompromised patients in Germany: a retrospective, observational Study on Health Insurance Data from 2021 to 2022
Source: Infection. 2025 Apr 11;53(5):1887–97. doi: 10.1007/s15010-025-02516-w (PMC12460495; doi:10.1007/s15010-025-02516-w)
Supplement: Supplementary file 1 — Supplementary file1 (DOCX 26 KB) [file 15010_2025_2516_MOESM1_ESM.docx]

Table S1: Operationalization of immunosuppressive diseases and therapies as well as COVID-19 diagnoses^a^

| **Group** | **Operator** | **ICD-10-Code** | **ATC-Code** | **OPS-Code** | **EBM-Code** |
| --- | --- | --- | --- | --- | --- |
| Autoimmune disorder under immunosuppressive treatment (including B cell depletion) | At least one diagnosis of | M05, M06, M30, M31.3, M31.5, M31.7, M32-M34, M35.2, D86, L40.0, G35, L10.0, N00.0-N00.7, N01.0-N01.7, N02.0-N02.7, N03.0-N03.7, N04.0-N04.7, N06.0-N06.7, N07.0-N07.7, K75.4 |  |  |  |
|  | And at least one of the following codes |  | L04AA, L04AB, L04AC, L04AD, L04AX, L01FA01, L01FA02, L01FA03 |  |  |
| CKD requiring dialysis | At least one diagnosis of | N18.4, N18.5 |  |  |  |
|  | And at least one of the following codes | Z49 |  |  | 13602, 13610, 13611, 04562, 04564, 40815, 40823, 40824, 40817-40819, 40826-40828 |
| Hepatic fibrosis and cirrhosis | At least one diagnosis of | K74, K70.3, K70.41, K70.42, K71.7, K72.1 |  |  |  |
| Cancer under therapy | At least one diagnosis of | C00-C97, D46, D47.1, D47.4 |  |  |  |
|  | And at least one of the following codes | Z94.80, Z94.81, D90 | L01 (except L01CH, L01CP), L04AA, L04AB, L04AC, L04AD, L04AX | 8-52, 8-53, 8-541 to 8-547, 8-549 | 17372, 25214, 25310, 25321, 25324-25328, 25330-25336 |
| Hematological malignancies under therapy | Diagnosis of | C81-C96 | L01 (except L01CH, L01CP), L04AA, L04AB, L04AC, L04AD, L04AX | 8-52, 8-53, 8-541 to 8-547, 8-549 | 17372, 25214, 25310, 25321, 25324-25328, 25330-25336 |
|  | At least one of the following codes | Z94.80, Z94.81, D90 |  |  |  |
| Organ Transplant and/or administration of mechanistic Target of Rapamycin (mTOR) and/or calcineurin inhibitors | At least one of the following codes | Z94.0-Z94.4, Z94.88 | L01EG, L04AD |  |  |
| Primary/secondary immunodeficiencies | At least one of the following codes | D80-D84 |  |  |  |
| Outpatient diagnosis for COVID-19 | Diagnosis of | U07.1!, *U09.9!, U10.9* |  |  |  |
|  | At least one of the following codes | R05, R06.0, R07.0, R07.1, R50.88, R50.9, R43, R51, M79.1, R63.0, R11, R10.1, R10.3, R10.4, A09, A08.3, B30.8, R59.0, R53, R45.3, R40.0, R42, R41.0, J00, J02.8, J04, J06, J12.8, J20.8, J21.8, J22, J80, J81, J96.0, R65.0, R65.1, B33.8, R57.2, A41.8, U10.9 |  |  |  |
| Hospitalization due to COVID-19 | Diagnosis of | U07.1!, *U09.9!, U10.9* |  |  |  |
|  | At least one of the following codes | R05, R06.0, R07.1, R50.88, R50.9, R51, R11, R40.0, R42, J12.8, J20.8, J21.8, J80, J81, J96.0, R65.0, R65.1, R57.2, A41.8, U10.9 |  | 8-70, 8-71, 8-72, 8-852 |  |

Abbreviations:

ATC: Anatomical Therapeutic Chemical Classification System, CD4+: Cluster of differentiation 4 positive, CKD: Chronic kidney disease, EBM: Einheitlicher Bewertungsmaßstab (uniform assessment criteria), ICD-10: 10th Revision of the International Statistical Classification of Diseases and Related Health Problems, OPS: Operationen- und Prozedurenschlüssel (Operation and procedure code)

Footnote:

^a^ A slightly different set of codes was used in 2022. Codes in italics were only used in 2022 evaluation
